# GC-MS-Based Metabolomics Study of Single- and Dual-Species Biofilms of *Candida albicans* and *Klebsiella pneumoniae*

**DOI:** 10.3390/ijms22073496

**Published:** 2021-03-28

**Authors:** Emilia Galdiero, Maria Michela Salvatore, Angela Maione, Elisabetta de Alteriis, Anna Andolfi, Francesco Salvatore, Marco Guida

**Affiliations:** 1 Department of Biology, University of Naples ‘Federico II’, via Cinthia, 80126 Naples, Italy; egaldier@unina.it (E.G.); angela.maione@unina.it (A.M.); dealteri@unina.it (E.d.A.); marco.guida@unina.it (M.G.); 2 Department of Chemical Sciences, University of Naples ‘Federico II’, via Cinthia, 80126 Naples, Italy; andolfi@unina.it (A.A.); frsalvat@unina.it (F.S.); 3 BAT Center—Interuniversity Center for Studies on Bioinspired Agro-Environmental Technology, University of Naples ‘Federico II’, Portici, 80055 Naples, Italy

**Keywords:** metabolomics, GC-MS, *Candida albicans*, *Klebsiella pneumoniae*, polymicrobial biofilms, interspecies interactions

## Abstract

*Candida albicans* and *Klebsiella pneumoniae* frequently co-exist within the human host as a complex biofilm community. These pathogens are of interest because their association is also related to significantly increased morbidity and mortality in hospitalized patients. With the aim of highlighting metabolic shifts occurring in the dual-species biofilm, an untargeted GC-MS-based metabolomics approach was applied to single and mixed biofilms of *C. albicans* and *K. pneumoniae*. Metabolomic results showed that among the extracellular metabolites identified, approximately 40 compounds had significantly changed relative abundance, mainly involving central carbon, amino acid, vitamin, and secondary metabolisms, such as serine, leucine, arabitol, phosphate, vitamin B6, *cyclo*-(Phe-Pro), trehalose, and nicotinic acid. The results were related to the strict interactions between the two species and the different microbial composition in the early and mature biofilms.

## 1. Introduction

*Candida* infection is a global threat for human health due to the high morbidity and mortality related to it in both developed and developing countries. The pathogenicity of *Candida* species is mediated by several virulence factors, including the ability to form a thick, organized, complex biofilm comprising blastospores, pseudo-hyphae, and hyphae, partially embedded in an extracellular polymeric matrix, on mucosal surfaces and medical devices. Different phases in the formation of *Candida* biofilms (adhesion, proliferation, filamentation, maturation, and dispersion) have been described [1].

*Candida albicans* is the most common species isolated in medically relevant biofilms. Hyphae are a characteristic feature of the *C. albicans* biofilm that contribute to its architectural stability. Furthermore, the *C. albicans* biofilm strengthens due to the production of an extracellular polymeric substance during its development, which contributes to its maturation. So, the established *C. albicans* biofilm is a complex structure that releases daughter cells able to disseminate to different niches and eventually develop into new biofilms [2].

Biofilm infections may be caused by a single microorganism or by a community of different species. These polymicrobial biofilms have been found in almost every niche in the human body [3]. The therapeutic management of infectious diseases due to the occurrence of polymicrobial biofilms is extremely difficult since the heterogeneity of species within mixed biofilms makes it difficult to assess the relevance and contribution of each individual species to pathogenesis and disease.

The complex structure of a polymicrobial biofilm allows stratification into spatially organized populations of the involved species. In this peculiar microenvironment, and during the different phases of biofilm formation, the products of microbial metabolism allow cells to communicate and share information. Therefore, interspecies interactions, ranging from synergistic relationships to competition among the different species in the biofilm, have been observed [4,5].

Bacteria are commonly associated with *C. albicans* in polymicrobial biofilms developed on medical devices. The interactions between *C. albicans* and bacterial species like *Staphylococcus epidermidis*, *Streptococcus pyogenes*, *Pseudomonas aeruginosa*, *Escherichia coli*, and *Bacillus subtilis* are well known and significantly high. In mixed biofilms of *C. albicans* and *Staphylococcus aureus*, a synergistic relationship between the two species was observed [6], while the interaction between *C. albicans* and *Salmonella typhimurium* was reported to be antagonistic because of the inhibition of *C. albicans* filamentation by the bacterium [7].

However, among the existing studies on mixed biofilm involving bacterial–fungal infections, there are only a few that deal with the interactions between *C. albicans* and *Klebsiella pneumoniae*.

*Klebsiella pneumoniae* is a Gram-negative opportunistic pathogen that can infect critically ill and immunocompromised patients, causing commonly healthcare-associated infections involving the lungs (e.g., pneumonia), the urinary tract, and the bloodstream. *K. pneumoniae* infections become progressively more difficult to treat due to both the antibiotic resistance and the hypervirulence of several clinical strains, so *K. pneumoniae* is one of the species listed as “priority pathogens” by the World Health Organization to stimulate research and development of new antibiotics [8,9].

Little is known about a statistically significant association between *K. pneumoniae* and *C. albicans* found in tracheal and mouth samples [10] and about the fact that their biofilms can colonize catheters and contribute to catheter-related septicemia [11]. Recently, it was reported that *K. pneumoniae* was able to form a multi-species biofilm together with *C. albicans* within a urinary catheter removed from a hospitalized patient [12].

Within our recent activity aiming to study in vitro models of single- and dual-species biofilm structures of *C. albicans* and *K. pneumoniae*, we demonstrated that both natural extracts from *Allium ursinum* and *Allium oschaninii* were able to inhibit and eradicate static and dynamic biofilms of the two species [13]. Furthermore, we reported the antimicrobial and antibiofilm activities of the synthetic peptides gH625-M and WMR-K toward these microbial models [14,15], confirming the importance of developing new strategies for dealing with polymicrobial biofilms. Further evidence of the effect of the synthetic peptides was obtained observing the metabolome alterations caused by the peptide WMR-K [15], but apart from that, not enough data are reported regarding the dynamic metabolic changes associated with the formation of a *C. albicans*/*K. pneumoniae* biofilm.

In fact, the whole group of metabolites reflects the physiological state of the microorganism, and therefore, metabolomics approaches are useful for the investigation of changes caused by culture development, the presence of active substances, or cultivar conditions. In particular, metabolic footprinting (i.e., the analysis of metabolites secreted and/or uptaken by cells from the culture medium) is a powerful tool that requires few steps, reducing the variability caused by sample handling, and for this reason, this approach is commonly used to highlight metabolomic changes [16,17].

We adopted an untargeted GC-MS-based metabolomics strategy to study the metabolic perturbations in single- and dual-species biofilms of *C. albicans* and *K. pneumoniae* due to the co-existence of these pathogens during biofilm development. This study represents a contribution to the knowledge of the formation process of mixed biofilms from the perspective of preventing the negative effects of microbial pathogen colonization.

## 2. Results and Discussion

The two species under examination (i.e., *C. albicans* and *K. pneumoniae*) developed single- and dual-species biofilms under the static experimental conditions adopted that were optimized, as described in our previous work [18].

**Figure 1:**
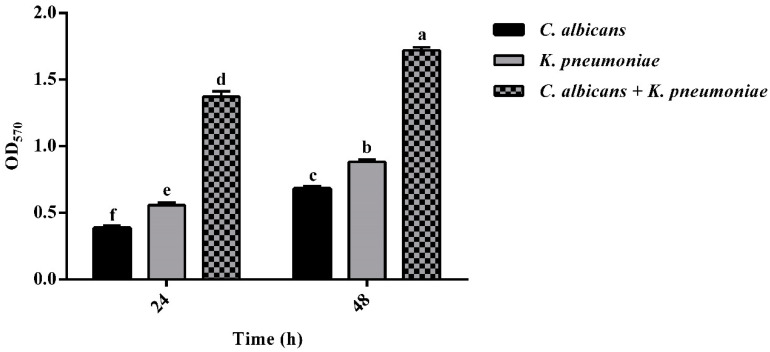
Quantification of biomass of single- and dual-species biofilm at 24 and 48 h; *n* = 3 ± SD; data with different letters (a–f) are significantly different (two-way ANOVA followed by Tukey’s post hoc test; *p* < 0.05). Values with dissimilar letters are significantly different from each other (*p* < 0.05). Values with the same letter are not significantly different (*p* > 0.05).

The adherent total biomass of each species was quantified by crystal violet (CV) staining after 24 and 48 h of incubation (Figure 1).

The *K. pneumoniae* single biofilm had a slightly higher biomass than the *C. albicans* single biofilm, whereas a significantly stronger fungal/bacterial structure was detected when the two species were allowed to adhere and proliferate in the mixed biofilm. Furthermore, the biomass in both single- and dual-species biofilms increased between 24 and 48 h, indicating that biofilm development continued when incubation was prolonged up to 48 h.

**Figure 2:**
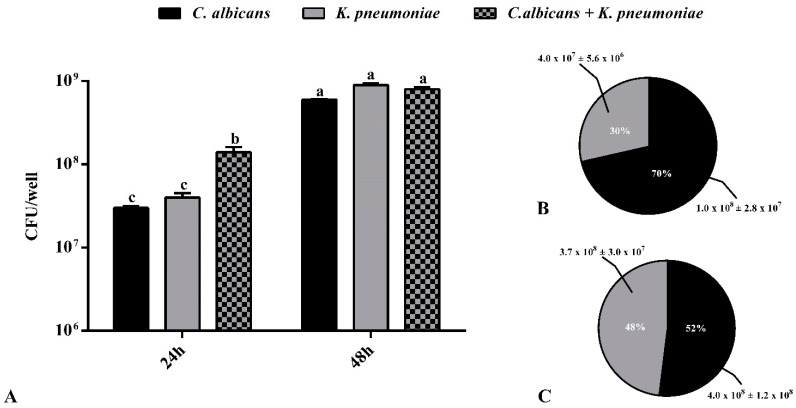
Viable cells in single- and dual-species biofilm at 24 and 48 h; *n* = 3 ± SD (**A**). Percentage composition and colony-forming units (CFU) per well ± SD (*n* = 3) of *Candida albicans* and *Klebsiella pneumoniae* in the dual-species biofilm at 24 h (**B**) and 48 h (**C**). Data with different letters (a–c) are significantly different (two-way ANOVA followed by Tukey’s post hoc test; *p* < 0.05). Values with dissimilar letters are significantly different from each other (*p* < 0.05). Values with the same letter are not significantly different (*p* > 0.05).

The same increasing trend between 24 and 48 h was observed when the number of viable cells of the two species, expressed as colony-forming units (CFU) per well, obtained from disrupted single- and dual-species biofilms, was considered (Figure 2A).

Interestingly, the cell composition in the dual-species biofilm significantly differed between 24 and 48 h (Figure 2B,C). The plate count on selective media of the cells recovered from the 24 h mixed biofilm clearly showed that it was mainly composed of *C. albicans*, whereas in the mature biofilm, the two species were in the same amount. The dominance of *C. albicans* in the 24 h mixed biofilm was also confirmed by the positive value of the competitive index (CI), which was tenfold higher compared to that calculated for the 48 h biofilm (0.4 vs. 0.04, respectively).

Actually, the starting advantage of *C. albicans* may be due to competition for the glucose-based medium and the favorable initial aerobic conditions; later, once the biofilm is established, and the environment gradually becomes more anaerobic, *K. pneumoniae* proliferation occurs until the two species equally co-exist and interconnect in the biofilm. It has been reported that the establishment of a *C. albicans* biofilm creates a hypoxic microenvironment, which enables the growth of both facultative and strict anaerobic bacteria [19].

The impact of each pathogen on the polymicrobial system was investigated using a GC-MS-based metabolic untargeted footprinting strategy, which focuses on the analysis of extracellular metabolites (i.e., on the excretion and uptake of metabolites from the culture medium). Within this framework, single planktonic and biofilm cultures and dual-species biofilm cultures of *C. albicans* and *K. pneumoniae* were examined.

The full set of GC-MS acquisitions comprised 36 observations (GC-MS chromatograms) segmented into six different conditions or classes (i.e., *C. albicans* planktonic and biofilm 24 h; *K. pneumoniae* planktonic and biofilm 24 h; and dual-species *C. albicans/K. pneumoniae* biofilms 24 and 48 h). Each class comprised three biological replicates, and GC-MS analysis of each biological replicate was replicated two times, giving six samples in each of the six classes.

Each single GC-MS acquisition was first presented to the Automated Mass Spectral Deconvolution and Identification System (AMDIS) program for deconvolution, component detection, and signal quantification [20]. Then, AMDIS results were presented to the SpectConnect program, which performs correspondence multigroup analysis of components across samples and creates a matrix (relative abundance (RA) matrix) with rows corresponding to observations and columns to metabolites grouped across samples [21]. Each cell of the RA matrix contains the relative abundance (RA) of a metabolite, encoded by the column label, in the observation specified by the row label.

The RA matrix lists only conserved metabolites. Technically, in the context of SpectConnect software, a conserved metabolite is one whose signal consistently persists in the replicated GC-MS chromatograms of at least one class (condition) and is said to define a clique.

The RA matrix created in the preprocessing step was then imported in MATLAB R2019b [22] for further processing with our in-house .m script.

**Figure 3:**
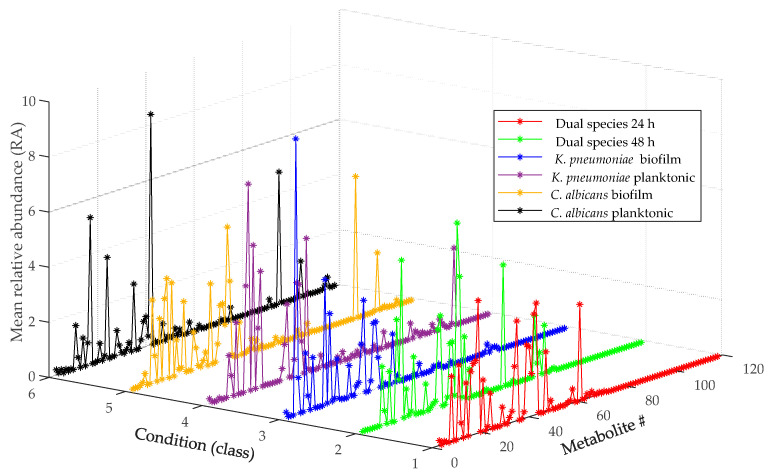
Graphical representation of metabolic footprinting data listed in the relative abundance (RA) matrix for the six conditions (classes) examined in this study.

The contents of the RA matrix are synthetically presented in Figure 3, where we can see a color-coded polyline corresponding to each condition. In the RA matrix, each metabolite in each class is represented by a 6 × 1 vector of RA values (derived from measurements on biological and technical replicates under the same experimental conditions). In Figure 3, points connected by the polyline represent the mean of the RA values in the vector associated to each metabolite in each class.

Please note that at this stage, metabolites had not yet been identified and were only differentiated on the basis of a code name or number (from 1 to 118 in Figure 3) and of the associated deconvoluted 70 eV electron impact (EI) mass spectrum. Nonetheless, inspection of Figure 3 shows that differences in metabolite levels exist between different classes.

To obtain an overview of the full dataset and visualize similarities and/or differences between observations, the whole RA matrix created in the preprocessing step was submitted to unsupervised multigroup principal component analysis (PCA).

**Figure 4:**
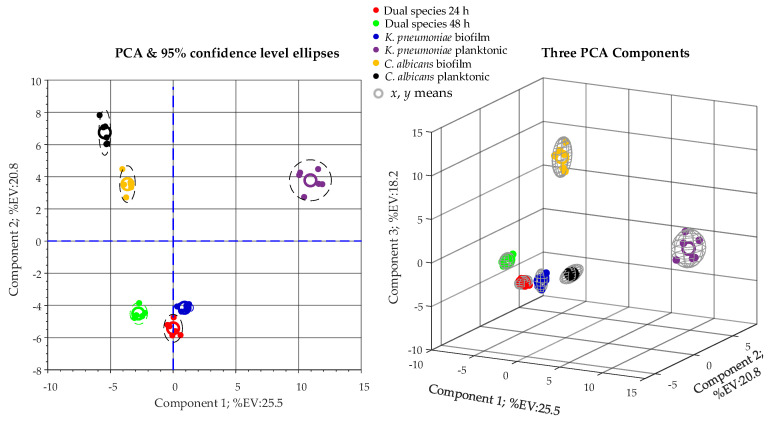
2D (**left**) and 3D (**right**) principal component analysis (PCA) score plots obtained from metabolomic profiles of all six cultural conditions (classes) under examination in this study.

Before PCA, to give equal weight to all metabolites (variables), data were mean-centered and unit variance (UV)-scaled by passing the data to the MATLAB zscore function. Finally, the centered and UV-scaled RA matrix was passed to the “pca” MATLAB function. Results of the untargeted multigroup PCA analysis are presented in the scores plot of Figure 4.

In Figure 4, we see a clear within-class grouping and between-class separation of observations. Because the PCA algorithm does not use class information (unsupervised), the score plot in Figure 4 certifies that there are real similarities between observations in the same class (i.e., grouping of bullets of the same color) and real differences in metabolite levels between classes (i.e., separation of bullets of different colors). In other words, whatsoever the PCA components might represent, the six cultural conditions considered in this study expose different metabolic patterns. Furthermore, the strict grouping of observations in the same class gives an indication of the repeatability of biological and technical replicates.

To interrogate the data for metabolites that are responsible for separation between classes, pairwise comparison of classes (conditions) is a more effective approach than multigroup comparisons.

From the six classes considered in this study, 15 pairs could be extracted and compared. Only a few of these pairwise comparisons, which were most relevant to unravelling metabolically significant differences, are revealed below. Pairwise class comparisons discussed in the following are synthesized in Table 1.

As can be seen in Table 1, pairwise class comparisons, at the multivariate level, have been performed by the widely employed supervised technique partial least-squares discriminant analysis (PLS-DA), in which observations are assigned ex ante to the class they belong to, and this class information is employed by the PLS-DA algorithm to construct a model that predicts this membership from the data.

Our MATLAB .m file has the logic to extract, from the RA matrix, data for any pair of classes (conditions) and to submit the extracted data to pairwise PLS-DA analysis, which is performed by passing the pertinent data to the MATLAB function plsregression.

In general, because the class membership of observations is known in advance, application of the PLS-DA technique results in better grouping of data in the same class and improved separation between classes with respect to PCA.

However, because PLS-DA tends to overfit the data, PLS-DA models need to be validated. The statistical significance of the PLS-DA models discussed below was assessed by the statistics R2X, R2Y, and Q2Y, which are reported for each pairwise comparison in Table 1. In particular, the high values of Q2Y (evaluated using mean squared errors from fivefold cross validation) indicate that the PLS models have a good predictive potential.

As a product of PLS-DA analysis, with each variable (metabolite) in the two compared classes, a variable importance in the projection (VIP) score, which indicates the importance of the variable in the model, is associated. Variables significantly influencing between class separation are associated with VIP scores of >1.

At the univariate level, to determine metabolites whose levels in the extracellular medium are significantly different between the two conditions, Student’s *t*-test is performed between the two 6×1 vectors of RA values representing each metabolite in the two compared conditions in the RA matrix. The statistical univariate test returns, for each metabolite, a *p*-value and a fold change (FC) calculated as the ratio between the means of the two compared vectors. At the 5% significance level, the FC is considered significantly different from 1 if the *p*-value < 0.05.

Finally, for each pairwise comparison, to each metabolite are attached a fold change (FC) value, a *p*-value (from Student’s *t*-test), and a VIP score (from pairwise PLS-DA analysis).

Because an untargeted approach is employed, only at the end of this process, to explore the biological significance of the untargeted analysis of the data, encoded metabolites are submitted to the identification procedure, which is essentially based on matching their deconvoluted 70 eV EI mass spectra with spectra in MS databases. As a general strategy, we also employed the Kovats retention index (RI), which is attributed to each metabolite on the basis of its chromatographic retention time [23], to enhance the confidence in the MS-spectrum-based identification and to filter the potential false-positive identifications generated by mass spectrum matching. After this, unidentified metabolites receive no further consideration and only identified metabolites are reported, unless an unidentified metabolite is considered exceptionally valuable to deserve further investigation.

First, we compared the metabolomic profiles of two culture modes (i.e., planktonic and biofilm) of each species (i.e., *C. albicans* and *K. pneumoniae*). The results showed that metabolomic profiles of both pathogen biofilms were characterized by higher levels of amino acids than in the respective planktonic cultures (see Supplementary Figures S1 and S2 and Table S1). This phenomenon was particularly evident in the biofilm of *C. albicans*, whose metabolome presented several upregulated amino acids, such as alanine (↑ fold = 1.91, VIP = 1.16), glutamic acid (↑ fold = 2.17, VIP = 1.21), glycine (↑ fold = 2.48, VIP = 1.25), isoleucine (↑ fold = 1.99, VIP = 1.23), lysine (↑ fold = 3.67, VIP = 1.13), methionine (↑ fold = 1.50, VIP = 1.25), phenylalanine (↑ fold = 1.91, VIP = 1.06), proline (↑ fold = 39.10, VIP = 1.26), serine (↑ fold = 1.50, VIP = 1.20), threonine (↑ fold = 2.16, VIP = 1.22), tryptophan (↑ fold = 2.40, VIP = 1.11), and valine (↑ fold = 2.18, VIP = 1.21). These findings are consistent with previous studies that reported upregulation of amino acids during biofilm formation [24,25,26]. A possible explanation can be that microorganisms metabolize nutrients, including amino acids, into tricarboxylic acid cycle (TCA) intermediates and their accumulation in biofilm cultures indicates less active metabolism. This result is also supported by the high level of phosphate (↑ fold ≈ 717, VIP = 1.04) in *C. albicans* biofilm culture, which is an essential nutrient required for cellular energy metabolism, and consequently, its accumulation can be explained by the inhibited energy metabolism. On the other hand, the increase in amino acids in the biofilm with respect to planktonic cultures might be the result of the activation of genes involved in their biosynthesis [24,25,26]. In fact, biofilm development is in general accompanied by the production of a large amount of extracellular matrix, which is, in part, due to the accumulation of carbohydrates and amino acids [26,27]. In this regard, the upregulation of amino acids in a biofilm might contribute to its formation, which plays a crucial role in the final architecture of the community.

**Figure 5:**
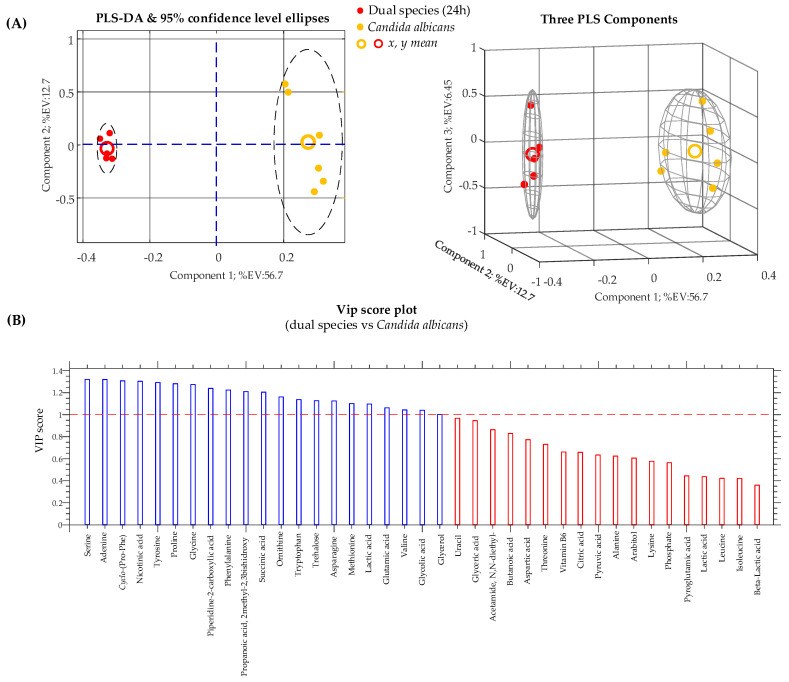
(**A**) Partial least-squares discriminant analysis (PLS-DA) score plots obtained from metabolomic profiles of a *Candida albicans* biofilm and a dual-species biofilm of *Candida albicans*/*Klebsiella pneumonia*. (**B**) Variable importance in the projection (VIP) chart of metabolites that contribute to separating the biofilm cultures. Metabolites significantly affecting between-class discrimination are the ones with VIP scores of >1.

**Figure 6:**
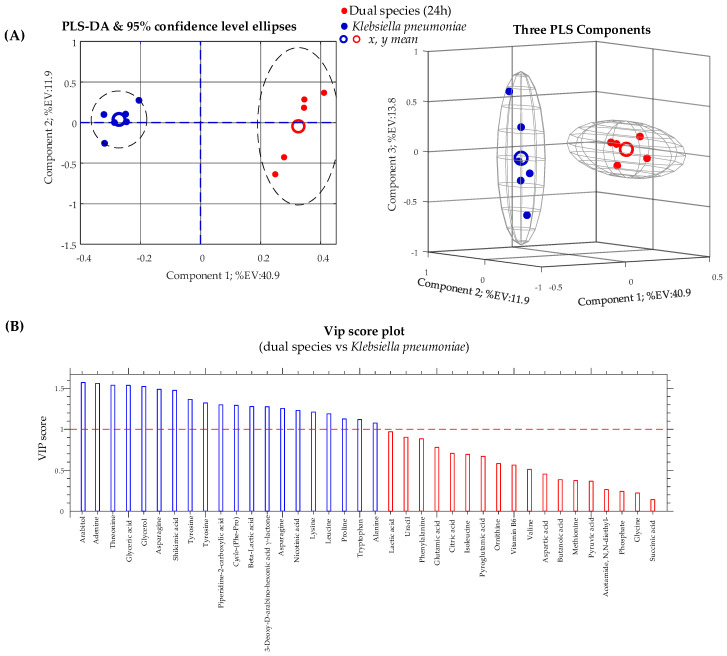
(**A**) Partial least-squares discriminant analysis (PLS-DA) score plots obtained from metabolomic profiles of a *Klebsiella pneumoniae* biofilm and a dual-species biofilm of *Candida albicans*/*Klebsiella pneumonia*. (**B**) Variable importance in the projection (VIP) chart of metabolites that contribute to separating the biofilm cultures. Metabolites significantly affecting between-class discrimination are the ones with VIP scores of >1.

Subsequently, we compared dual-species *C. albicans*/*K. pneumoniae* biofilm culture with single-species *C. albicans* and *K. pneumoniae* biofilm cultures after 24 h of incubation. PLS-DA results for these two pairwise comparisons are presented, respectively, in Figure 5 and Figure 6, in the form of 2D and 3D score plots (Figure 5A and Figure 6A) and bar plots (Figure 5B and Figure 6B), exposing VIP values for identified conserved metabolites. In addition, VIP scores, *p*-values, and fold change (FC) for each identified conserved metabolite are revealed in Table 2.

From Figure 5 and Table 2, it is clear that serine is the most important metabolite that contributes to the distinction between the *C. albicans* biofilm and the dual-species community. In particular, it can be seen that the serine level decreased by about 300 times in the dual-species biofilm with respect to the single-species *C. albicans* biofilm.

Please note that this high serine fold change is the result of the fact that no serine signal is detected for the dual-species biofilm and missing RA values are substituted by data imputation, as explained in the Materials and Methods section. In other words, serine is not a conserved (detected) metabolite in the *C. albicans*/*K. pneumoniae* biofilm class.

On the other hand, serine is also not a conserved metabolite in the *K. pneumoniae* biofilm class, and therefore, it is completely excluded when dual-species *C. albicans*/*K. pneumoniae* and single-species *K. pneumoniae* biofilms are compared. This fact is indicated by a hyphen in Table 2.

Since serine is present as a nutrient in the tryptic soy broth (TSB) culture medium, it is obvious that the consumption of serine in the fungal/bacterial biofilm is mainly or exclusively due to *K. pneumoniae*. This effect of the bacterial component on the serine level of the dual-species biofilm is analogous to the 125 times decrease in the serine level in the *P. mirabilis/C. albicans* biofilm with respect to the single-species *C. albicans* biofilm, observed by Kart et al. [24]. This point of view is also supported by the data obtained in our previous work, which investigated the effect of the antimicrobial peptide WMR-K on dual-species *C. albicans*/*K. pneumoniae* biofilm culture. In fact, it was observed that the presence of this synthetic peptide influences the level of serine due to its antibacterial activity toward *K. pneumoniae*, and as a consequence, there was a higher level of serine in the treated dual-species biofilm than in the untreated one [15].

Similarly, but to a lower extent, trehalose decreased in the mixed biofilm, indicating the negative effect of the presence of *K. pneumoniae* in the synthesis of a molecule exclusively produced by the fungus.

Further, the preferential accumulation of adenine, ornithine, phenylalanine, tyrosine, and tryptophan in the dual-species biofilm compared to the single-species *Candida* biofilm (Table 2) could be ascribed to the active proliferation of the mixed consortium that forms a biofilm with a significantly higher biomass (see Figure 1).

In addition to consuming serine, *K. pneumoniae* also appears, from data in Table 2, to consume leucine. In fact, leucine, which is present in the TSB medium, is not a conserved metabolite in the *K. pneumoniae* biofilm class. This is not surprising because bacteria not only can assimilate leucine through specific uptake transport systems but also have a catabolic pathway specific for this amino acid [28]. However, unlike serine, leucine is present in the dual-species biofilm class and the level of leucine in the dual-species biofilm is not significantly different from its level in the *C. albicans* biofilm. This implies that although leucine itself seems indifferent to *C. albicans*, the presence of the fungus mitigates the uptake of leucine by *K. pneumoniae*. Therefore, inhibition of the specific bacterial transport system for leucine might be one factor that contributes to the starting advantage of *C. albicans* over *K. pneumoniae* during the initial and intermediate phases of the process of biofilm formation in the dual-species culture (see Figure 2).

Another interesting metabolic effect of the co-existence of *C. albicans* and *K. pneumoniae* can be unpicked by the observation of the relative abundance of arabitol (a sugar alcohol) in the dual-species biofilm compared to the single-species biofilm. First, as can be seen from Figure 6B, arabitol is the most important metabolite contributing to the differentiation between the dual-species biofilm and the *K. pneumoniae* biofilm. Arabitol is not a conserved metabolite in the *K. pneumoniae* biofilm class, but it is a conserved metabolite in the dual-species biofilm. Consequently, arabitol is markedly upregulated in the bacterial/fungal biofilm culture with respect to the single-species bacterial biofilm culture (↑ fold ≈ 883, VIP = 1.57). Second, the level of arabitol in the dual-species biofilm is not significantly different from its level in the *C. albicans* single-species biofilm culture (=fold ≈ 1.3, VIP = 0.6; see Table 2). The most parsimonious interpretation of these facts is that arabitol is produced and excreted in the medium by *C. albicans*. In fact, it has been reported [29,30] that yeasts, and among them several *Candida* species, are capable of producing arabitol from glucose and our data confirm this fact. As a result, from our data, it appears that *C. albicans* is capable of maintaining glucose metabolism in the dual-species biofilm at the same level as in the single-species biofilm, reducing this resource for *K. pneumoniae*, and this might be an additional factor contributing to its success in the early phase of dual-species biofilm development (see Figure 2).

In addition, glycerol’s preferential accumulation in the dual-species biofilm (Table 2) can be ascribed to the dominance of *C. albicans* in the 24 h biofilm.

In this study, a result that should be emphasized concerns the metabolites involved in microbial virulence and pathogenicity, i.e., nicotinic acid and *cyclo*-(Phe-Pro), which show significant changes in their relative abundance. In fact, nicotinic acid is a NAD^+^ precursor that influences the adhesion of *Candida* species to different biomaterials and epithelia [31,32], and *cyclo*-(Phe-Pro) is a secondary metabolite important in cell-to-cell communication by bacteria and fungi [33,34,35,36]. As can be seen from Table 2, these metabolites were downregulated in the dual-species biofilm culture, and even if some mechanisms are not known yet and would need further investigation, these metabolites might play a role in the context of microbial interactions within the biofilm.

**Figure 7:**
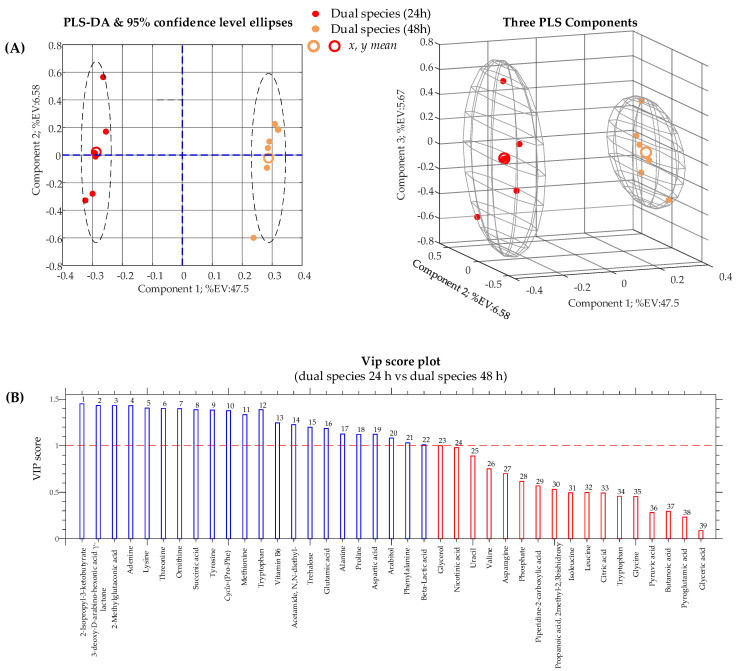
(**A**) Partial least-squares discriminant analysis (PLS-DA) score plots obtained from metabolomic profiles of the dual-species biofilm of *C. albicans*/*K.* *pneumoniae* after an incubation period of 24 and 48 h. (**B**) Variable importance in the projection (VIP) chart of metabolites that contribute to separating the biofilm cultures. Metabolites significantly affecting between-class discrimination are the ones with VIP scores of >1.

In sessile cultures, 24 h is the time required for cells to form a substantial biofilm. This time was extended to 48 h to obtain a mature biofilm and, eventually, to unravel interesting metabolic evolutions of the dual-species biofilm culture in time. To achieve this goal, the data obtained for *C. albicans*/*K. pneumoniae* biofilm at 24 h were compared with the ones obtained after 48 h of incubation. The PLS-DA score plots presented in Figure 7A show a clear discrimination between the two conditions, and the VIP score bar plot in Figure 7B reveals that several identified metabolites play a role in class separation.

Several nutrients present in the culture medium were downregulated in the dual-species biofilm culture after 48 h of incubation, with respect to the 24 h culture (see Supplementary Table S2). Downregulation of nutrients after 48 h of incubation is an expected phenomenon since, obviously, nutrients are consumed to maintain the growth of the developing culture and/or for the survival of the microorganisms. For this reason, downregulated metabolites in the biofilm culture after 48 h of incubation are relatively uninteresting (although they may substantially contribute to the discrimination between the two classes).

For instance, several amino acids, such as alanine (↓ fold = 1.27, VIP = 1.126), glutamic acid (↓ fold = 1.22, VIP = 1.186), lysine (↓ fold = 6.42, VIP = 1.406), methionine (↓ fold = 1.58, VIP = 1.333), and tryptophan (↓ fold = 4.37, VIP = 1.388), were downregulated in the mature (48 h) dual-species biofilm with respect to the dual-species biofilm at 24 h. This is not surprising, considering that some amino acids represent a source of nutrients and can be metabolized into TCA intermediates and enter this cycle.

On the contrary, upregulated metabolites in the mature phase (48 h) of the dual-species biofilm are more likely to provide useful information, since, in abstract, they are likely to derive from the microorganism’s biosynthetic activity.

Between upregulated metabolites in the 48 h biofilm culture, vitamin B6 (↑ fold = 4.35, VIP = 1.246) and trehalose (↑ fold = 3.08, VIP = 1.199) can be unpicked.

Both vitamin B6 and trehalose (a nonreducing sugar containing two glucose subunits) are considered cellular protectors against nutritional and/or environmental stress [29,37,38,39]. For instance, upregulation of trehalose has been observed in *C. albicans* biofilms (single and polymicrobial) treated with antimicrobial agents [15,40].

However, in fungi, trehalose acts primarily as a reserve of carbohydrates. In particular, trehalose is produced from glucose when this sugar is abundant in the environment, and is consumed when the environment is depleted of glucose (trehalose is degraded into two glucose molecules) [38].

Our data confirm that trehalose is produced by *C. albicans* because it is only present in samples containing the fungal species and it is altogether absent in *K. pneumoniae* single-species biofilm culture.

Nevertheless, in opposition to what is expected (i.e., accumulation of trehalose in the early phase of biofilm development and trehalose depletion in the mature phase), we found that trehalose was upregulated in the mature biofilm (48 h). We conclude that accumulation, by *C. albicans*, of trehalose in the mature biofilm is not connected to its primary function as reservation of carbohydrates but, rather, to its role as a cell protector against an unfavorable environment. This hypothesis is supported by the fact that upregulation of trehalose in the mature biofilm takes place contextually to the upregulation of vitamin B6.

As is well known, the accumulation of trehalose in fungi appears to be associated with periods of a reduced growth rate [41]. Therefore, accumulation of trehalose may be caused by the competition between *C. albicans* and *K. pneumoniae* in the mature phase of the biofilm and might be one factor contributing to the decline, from 70% (in the 24 h biofilm) to 52% (in the 48 h biofilm), of the percentage of *C. albicans*-viable cells in the dual-species biofilm culture (see Figure 2).

## 3. Materials and Methods

### 3.1. Strains Cultivation

*Candida albicans* ATCC 90028 and *Klebsiella pneumoniae* ATCC 10031 were kept on Sabouraud dextrose agar and trypticase soy agar (TSA), respectively, and grown in tryptic soy broth (TSB) with and without 1% glucose, respectively. Planktonic cells of each strain were cultured in Erlenmeyer flasks containing 25 mL of the proper medium, starting from an adequate aliquot of a pre-culture. Cultivations were carried out at 37 °C for 24 h under shaking conditions (200 rpm).

For mono- and polymicrobial biofilm formation, a single colony of each microbial strain was inoculated in TSB broth with/without glucose and incubated at 37 °C under shaking conditions overnight, then washed twice using sterile phosphate-buffered saline (PBS), and adjusted to 10^6^ cells·mL^−1^ in the respective culture medium.

### 3.2. Development and Quantification of Mono- and Polymicrobial Biofilms

Mono- and polymicrobial biofilms were developed with a modified protocol previously described by de Alteriis et al. [18]. For the dual-species biofilm, equal volumes of each strain suspension (10^6^ cells·mL^−1^) were mixed in TSB with glucose to achieve a ratio of 1:1. Briefly, 100 μL of mono- or dual-species cell suspensions were added to the wells of a flat-bottomed polystyrene 96-well microplate and incubated at 37 °C for 24 or 48 h to allow biofilm formation. After the incubation period, non-adherent cells were carefully withdrawn and the wells were gently washed with PBS. Then, the microplate was dried at 65 °C to fix the biofilms, stained with crystal violet (CV) (0.2% *v*/*v*), and incubated for 10 min at room temperature. After CV removal, the microplate was rinsed once with PBS and dried at 65 °C for 60 min. Biofilm formation was quantified by eluting the CV fixed to the biofilm in 33% glacial acetic acid, and the absorbance of each well was measured as optical density at 570 nm (OD_570nm_) using a microplate reader (SYNERGY H4 BioTek).

### 3.3. Quantification of Viable Cells in the Biofilms

The quantification of viable cells of each strain in single- or dual-species biofilms was determined using the plate-counting technique on selective media. For removal of sessile cells of the biofilms, sterile pipette tip scraping of the bottom of the well followed by repeated pipetting was performed. Cells recovered from the wells were serially diluted in PBS and plated on TSA (with 1 μg·mL^−1^ of amphotericin B) and rose bengal (with 20 μg·mL^−1^ of chloramphenicol) agar plates to count colony-forming units (CFU) for *K. pneumoniae* and *C. albicans*, respectively. Plates were incubated at 37 °C for 48 h. The values obtained were expressed as CFU per well. Assays were performed in triplicate.

The competitive index (CI), defined as the *C. albicans/K. pneumoniae* ratio within the output sample (CFU in the biofilm at 24 or 48 h) divided by the corresponding ratio in the inoculum (CFU in the inoculum), was calculated according to Ballen et al. [42] using the formula

$$\mathrm{CI}=\mathrm{Log}\left[\frac{\left(\mathrm{CFU}\;C.\;albicans\right)\;\mathrm{output}\;\times \left(\mathrm{CFU}\;K.\;pneumoniae\right)\;\mathrm{input}}{\left(\mathrm{CFU}\;K.\;pneumoniae\right)\;\mathrm{output}\;\times \;\left(\mathrm{CFU}\;C.\;albicans\right)\;\mathrm{input}}\right],$$

where CI = 0 indicates equal competition between species; CI > 0 indicates a competitive advantage for *C. albicans*; and CI < 0 indicates a competitive advantage for *K. pneumoniae*.

### 3.4. Metabolomic Analysis

#### 3.4.1. Sample Preparation

For analysis of extracellular metabolites from the planktonic cultures of *C. albicans* and *K. pneumoniae*, 100 μL of each culture was collected after 24 h of incubation and centrifuged (5000× *g*, 10 min).

For analysis of extracellular metabolites of the 24 and 48 h single- and dual-species biofilms, the liquid medium (100 μL) was carefully collected from the wells of the microplate, transferred into Eppendorf tubes, and centrifuged (5000× *g*, 10 min).

Then, supernatants from both planktonic cultures and biofilms were dried with a stream of nitrogen, and the residues were treated with *N*,*O*-bis(trimethylsilyl)-trifluoroacetamide (BSTFA) (Fluka, Buchs, Switzerland), as previously described [43]. Each class comprised three biological replicates, and GC-MS analysis of each biological replicate was replicated two times.

#### 3.4.2. GC-MS Analysis

Trimethylsilyl derivatives were analyzed using an Agilent 6850 GC instrument (Milan, Italy) coupled to an Agilent 5973 Inert MS. Then, 2 μL of each sample was injected in splitless mode into an HP-5MS capillary column ((5%-phenyl)-methylpolysiloxane stationary phase). The injection temperature was 250 °C, and the temperature ramp raised the column temperature from 70 °C to 280 °C: 70 °C for 1 min, 10 °C·min^−1^ until the column temperature reached 170 °C, and 30 °C·min^−1^ until the column temperature reached 280 °C. Subsequently, it was held at 280 °C for 5 min. Helium was used as a carrier gas at a flow rate of 1 mL·min^−1^. The solvent delay was set to 4 min. Measurements were performed under electron impact (EI) ionization (70 eV) in full scan mode (*m*/*z* 29–550) at a frequency of 3.9 Hz. The EI ion source and quadrupole mass filter temperatures were kept, respectively, at 200 and 250 °C.

#### 3.4.3. Data Processing and Statistical Analysis

GC-MS data were deconvoluted using the National Institute of Standards and Technology (NIST) program Automated Mass Spectral Deconvolution and Identification System (AMDIS) [20], and then the conserved metabolites across each biological and technical replicate were listed and tracked using SpectConnect [21]. Technically, in the context of SpectConnect software, a conserved metabolite is one that consistently persists in replicate samples (at least in 75% of the observations in one class or condition). Each metabolite’s relative abundance (RA) was auto-scaled, and the created dataset (RA matrix) was then submitted to multivariate statistical analyses, such as principal component analysis (PCA) and partial least-squares discriminant analysis (PLS-DA), which were performed with our in-house .m script in MATLAB R2019b (Mathworks, Natick, MA, USA) [22].

The principal advantage of PLS-DA in the present context is that the algorithm allows one to readily unpick variables (metabolites) that are most important for discrimination between classes. In fact, for each variable, a variable importance in the projection to latent variables (VIP) score can be calculated, which indicates the statistical significance of each compound (variable) in the PLS-DA model. Therefore, detected metabolites can be ranked based on their VIP scores, which helps in the process of biomarker selection. In general, the importance of variables for the PLS-DA model decreases as their VIP scores decrease, and only variables with VIP scores of >1 are considered to contribute significantly to the discrimination between the compared classes.

After multivariate analysis, pairwise comparisons of conditions are finalized by comparing metabolites in the two defined classes, one by one, using a univariate statistical test. This allows one to determine the metabolites whose levels in the extracellular medium are significantly different between the two conditions. Within this framework, for each conserved metabolite, two arrays of RA values (one array for each of the two compared conditions) are extracted from the RA matrix and passed to the ttest2 MATLAB function. This function compares the two vectors using unpaired Student’s *t*-test and returns, between other statistics, a *p*-value. At the 5% significance level, the two vectors are considered to have unequal means if the returned *p*-value is lower than 0.05. After this, for each metabolite, a fold change (FC) value is calculated from the two compared vectors. In abstract, a fold change is defined as the ratio of the concentrations of the metabolite in the two classes, but in an untargeted approach, the FC is evaluated as the ratio of the RA averages in the two compared vectors. The FC value attached to a metabolite during univariate data analysis is considered significantly different from 1 if the *p*-value associated with the metabolite from the above-described Student’s *t*-test is lower than 0.05.

Interestingly, we find that Student’s *t*-test *p*-values are strictly correlated to PLS-DA VIP scores. In fact, *p*-values decrease as VIP scores of metabolites increase, and almost invariably VIP scores > 1 translate into *p*-values < 0.05.

As noticed above (see Figure 3), the RA matrix created by SpectConnect from the 36 observations considered in this study encodes 118 metabolites. When comparing two defined conditions, we first extract from the 36 × 118 RA matrix a 12 × 118 submatrix representing the two selected conditions. The number of columns in the submatrix is then reduced by cancelling out columns with all elements equal to zero (because such columns encode metabolites that are not detected (not conserved) in any sample of the two compared classes and, by consequence, are irrelevant for the pairwise class comparison). Pairwise class comparisons are then based on a 12 × *k* submatrix in which the number of encoded metabolites, *k*, depends on the two compared conditions.

In the 12 × *k* submatrix, there are typically several zeros or missing elements corresponding to metabolites whose signals were not detected in a particular sample and to which RA = 0 is nominally assigned. Obviously, although possible, it is not warranted that a metabolite be altogether absent in a sample because its signal was undetected. In fact, a number of phenomena that take place during data acquisition (among others, noise and column bleeding) could obscure a low signal. Thus, it is reasonable to adopt the view that undetected signals actually are signals that fall under a minimum threshold. In the present work, we set the threshold equal to the minimum RA value for encoded metabolites in each chromatogram (i.e., to the minimum RA value in each row of the 12 × *k* submatrix). Then, each zero element eventually present in the 12 × *k* submatrix is substituted by a fraction of the corresponding threshold value. This is a prudent and conservative strategy of data imputation that, in general, will produce a fold change lower than the actual value, and this avoids overestimation of the role of a metabolite in the discrimination between classes.

For instance, when we compare the *C. albicans*/*K. pneumoniae* 24 h biofilm with *C. albicans* biofilm, the extracted RA submatrix has dimensions 12×81, implying that only 81 encoded metabolites are conserved in at least one of the two compared conditions. In the 12×81 submatrix, there are 136 elements (out of 972; about 14%) that are missing and that are substituted with a fraction of the corresponding threshold value.

In some cases, it may happen that a metabolite is not detected or is absent in all samples of one of the two compared conditions. This implies that during univariate analysis, one of the two compared vectors is a vector of all zeros. In such a case, data imputation is especially important to avoid distortion of the results of the univariate statistical test and the production of infinity fold change. For instance, as we have already noticed above, this is the case with serine when the *C. albicans*/*K. pneumoniae* 24 h biofilm is compared with the *C. albicans* biofilm class. The 6×1 vector representing serine in the dual-species class is a vector of all zeros that is substituted with the vector {0.0052, 0.053, 0.0046, 0.0052, 0.0046, 0.0023} according to the above data imputation strategy.

Metabolites were identified by comparing their deconvoluted EI mass spectra at 70 eV with spectra of known substances present in the NIST 14 mass spectral library [44] and the Golm metabolome database [45,46]. Furthermore, the identification was supported by the Kovats retention index (RI) calculated for each analyte by the Kovats equation using the standard *n*-alkane mixture in the range C7-C40 (Sigma-Aldrich, Saint Louis, MO, USA). The combined strategy of supporting a metabolite’s identification both by mass spectrum matching and retention index matching is very effective not only to enhance confidence in the identification but also to filter false-positive identifications generated by spectrum matching due to the fact that different compounds (e.g., isomers or compounds with similar structures) might have similar mass spectra. However, this strategy has two fundamental limitations. The first limitation depends on the fact that retention indices are empirical parameters and, by consequence, are subject to variations, especially related to the nature of the chromatographic stationary phase and the specific temperature program. Thus, when matching retention indices to confirm a putative compound’s identity, it is necessary to consider their variability. It is believed that variations in the temperature-programmed Kovats index of the same compound on standard semi-polar and polar phases may reach up to 25 index units [47]. Therefore, in our strategy, we generally consider that identification based on spectrum matching is confirmed if the difference of the RI experimentally determined by us is within ±15–25 index units from the retention indices reported in databases or in the literature, respectively, for a stationary phase identical and similar to that employed in this study (i.e., (5%-phenyl)-methylpolysiloxane) (see Table S3). Because of this limitation, we derogated from the above rule in two cases, as can be seen from Table S3, where an unknown mass spectrum matches to a high degree the library spectrum and the probability of a false positive is low or negligible. The second limit is related to the obvious fact that for uncommon compounds, no retention indices are reported. For instance, in Table S3, this is the case for 4 of 43 compounds.

## 4. Conclusions

In this study, biofilms formed by the major fungal species involved in polymicrobial-biofilm-based infections, *Candida albicans*, and the emergent pathogen *Klebsiella pneumoniae* were examined by integrating biological data on biomass and cell viability of single- and dual-species biofilms with a GC-MS-based metabolomics approach to exploring metabolic changes characterizing the dynamics of dual-species biofilm development with respect to single-species biofilm and planktonic control cultures.

Our biological data reveal that the biomass and cell composition of the fungal/bacterial biofilm significantly differ from those of the single-species biofilm after 24 h of incubation. Furthermore, changes are observed in the cell composition of the dual-species biofilm between 24 and 48 h of incubation. In fact, the starting advantage of *C. albicans* gained in the early phases of mixed biofilm formation is lost due to the proliferation of *K. pneumoniae*, which occurs until the two species equally co-exist in the biofilm.

The analysis of metabolic data demonstrates a general upregulation of amino acids in biofilms with respect to planktonic cultures. However, upregulation of amino acids observed in early *C. albicans*/*K. pneumoniae* biofilms is fated to decrease during biofilm development.

Metabolic data suggest that the observed prevalence of *C. albicans* in the early phase of the dual-species biofilm culture might be due to the capacity of the fungus to oppose the growth of *K. pneumoniae* by maintaining active glucose metabolism (as demonstrated by upregulation of arabitol) and to reduce bacterium uptake of leucine from the medium, possibly by the inhibition of the specific bacterial transport system for this amino acid.

Probably, the most evident demonstration that strict interactions exist between the two microbes in the fungal/biofilm culture is that stress-related metabolites produced by *C. albicans* (i.e., vitamin B6, trehalose) are more abundant in the mature biofilm. The costs of the biosynthesis of these cell protection metabolites in later phases of biofilm development are a viable explanation for the observed decline in *C. albicans* prevalence in the mature dual-species biofilm.

Therefore, biological and metabolomic results indicate that strict interactions exist between the two microbes that differentiate the *C. albicans*/*K. pneumoniae* biofilm from single-species biofilms and metabolic data explain the observed differences in the cell composition between the early fungal/bacterial biofilm and the mature biofilm. Consequently, these data are particularly relevant because they might help to figure out strategies to reduce or combat the infections caused by *C. albicans*/*K. pneumoniae,* a consortium of microbial species that so far has not been studied in detail, notwithstanding its potential pathogenicity.

## Figures and Tables

**Table 1:** Relevant information about pairwise class (conditions) comparisons discussed in the text. PLS-DA: partial least-squares discriminant analysis.

Pairwise Comparisons	Univariate Statistical Test	Multivariate Analysis	R2X	R2Y	Q2Y
(*C. albicans* planktonic) vs. (*C. albicans* biofilm)	Student’s *t*-test	PLS-DA	0.7287	0.9996	0.9876
(*K. pneumoniae* planktonic) vs. (*K. pneumoniae* biofilm)	Student’s *t*-test	PLS-DA	0.6749	0.9983	0.9663
(*C. albicans*/*K. pneumoniae* biofilm 24 h) vs. (*C. albicans* biofilm 24 h)	Student’s *t*-test	PLS-DA	0.7348	0.9994	0.9803
(*C. albicans*/*K. pneumoniae* biofilm 24 h) vs. (*K. pneumoniae* biofilm 24 h)	Student’s *t*-test	PLS-DA	0.6368	0.9952	0.9261
(*C. albicans*/*K. pneumoniae* biofilm 24 h) vs. (*C. albicans*/*K. pneumoniae* biofilm 48 h)	Student’s *t*-test	PLS-DA	0.5975	0.9988	0.9988

**Table 2:** Identified metabolites from metabolomic analysis of the dual-species biofilm culture of *C. albicans*/*K. pneumoniae* compared with the single-species biofilm of *C. albicans* and *K. pneumoniae*.

	Dual Species vs. *C. albicans*			Dual Species vs. *K. pneumoniae*		
Name	VIP Score	*t*-Test *p*-Value	Fold Change	VIP Score	*t*-Test *p*-Value	Fold Change
Acetamide, N,N-diethyl- (RI: 1045)	0.864	3.10 × 10^−2^	=2.24	0.264	6.70 × 10^−2^	=1.13
Adenine, TMS (RI: 1890)	1.320	2.86 × 10^−9^	↑ 25.63	1.561	3.12 × 10^−9^	↑ 19.85
Alanine, 2TMS (RI: 1124)	0.623	1.76 × 10^−1^	=1.15	1.076	2.16 × 10^−2^	↑ 1.24
Arabitol, 5TMS (RI: 1750)	0.606	1.78 × 10^−1^	=1.28	1.573	4.22 × 10^−13^	↑ 883.44
Asparagine, 3TMS (RI: 1687)	1.125	2.56 × 10^−3^	↓ 1.24	1.490	1.26 × 10^−7^	↓ 1.73
Aspartic acid, 3TMS (RI: 1540)	0.773	6.12 × 10^−2^	=1.59	0.454	3.94 × 10^−1^	=1.16
*Beta*-lactic acid, 2TMS (RI: 1156)	0.359	4.38 × 10^−1^	=1.17	1.278	2.32 × 10^−3^	↑ 1.76
Butanoic acid, 3TMS (RI: 1425)	0.829	4.17 × 10^−2^	=2.24	0.385	5.92 × 10^−1^	=1.03
Citric acid, 4TMS (RI: 1844)	0.659	1.28 × 10^−1^	=1.46	0.706	1.73 × 10^−1^	=1.58
*Cyclo*-(Phe-Pro) (RI: 2434)	1.308	5.26 × 10^−8^	↓ 8.84	1.293	1.87 × 10^−3^	↓ 4.51
3-Deoxy-D-arabino-hexonic acid γ-lactone, 3TMS (RI: 1797)	–			1.276	2.37 × 10^−3^	↓ 1.30
Glutamic acid, 3TMS (RI: 1638)	1.062	3.35 × 10^−3^	↑ 1.33	0.778	1.28 × 10^−1^	=1.12
Glyceric acid, 3TMS (RI: 1346)	0.946	1.76 × 10^−2^	=1.34	1.538	1.73 × 10^−7^	↓ 4.81
Glycerol, 3TMS (RI: 1290)	1.002	7.48 × 10^−3^	↓ 1.33	1.524	1.17 × 10^−6^	↑ 644.01
Glycine, 2TMS (RI: 1136)	1.274	3.46 × 10^−6^	↓ 1.83	0.223	9.37 × 10^−1^	=1.01
Glycolic acid (2TMS) (RI: 1100)	1.040	4.75 × 10^−3^	↓ 2.15	–		
Isoleucine, 2TMS (RI: 1307)	0.421	3.56 × 10^−1^	=1.60	0.694	1.82 × 10^−1^	=1.60
Lactic acid, 2TMS (RI: 1083)	0.437	3.42 × 10^−1^	=10.26	0.971	4.57 × 10^−2^	=7.38
Leucine, 2TMS (RI: 1286)	0.422	3.50 × 10^−1^	=1.32	1.189	7.00 × 10^−3^	↑ 523.57
Lysine, 3TMS (RI: 1722)	0.576	1.98 × 10^−1^	=1.23	1.212	6.35 × 10^−3^	↑ 1.41
Methionine, 2TMS (RI: 1536)	1.101	1.70 × 10^−3^	↓ 1.21	0.374	7.28 × 10^−1^	=1.02
Nicotinic acid, TMS (RI: 1304)	1.304	1.22 × 10^−7^	↓ 2.51	1.231	4.39 × 10^−3^	↓ 1.36
Ornithine, 3TMS (RI: 1632)	1.161	4.61 × 10^−4^	↑ 4.34	0.583	2.86 × 10^−1^	=1.20
Phenylalanine, 2TMS (RI: 1647)	1.224	6.09 × 10^−5^	↑ 1.26	0.884	8.34 × 10^−2^	=1.07
Phosphate, 3TMS (RI: 1297)	0.564	2.12 × 10^−1^	=1.48	0.242	7.02 × 10^−1^	=1.11
Piperidine-2-carboxylic acid, 2TMS (RI: 1624)	1.239	3.40 × 10^−5^	↓ 2.69	1.299	1.78 × 10^−3^	↓ 1.38
Proline, 2TMS (RI: 1314)	1.282	1.88 × 10^−6^	↓ 11.44	1.127	1.34 × 10^−2^	↓ 3.60
Propanoic acid, 2methyl-2,3bishidroxy, 3TMS (RI: 1336)	1.210	1.08 × 10^−4^	↓ 5.16	–		
Pyroglutamic acid, 2TMS (RI: 1546)	0.444	3.28 × 10^−1^	=1.23	0.669	2.06 × 10^−1^	=1.08
Pyruvic acid, 2TMS (RI: 1108)	0.633	1.67 × 10^−1^	=2.91	0.367	5.47 × 10^−1^	=1.19
Serine, 3TMS (RI: 1375)	1.320	1.49 × 10^−9^	↓ 307.99	–		
Shikimic acid, 4TMS (RI: 1951)	–			1.476	1.71 × 10^−5^	↓ 2.98
Succinic acid, 2TMS (RI: 1322)	1.205	1.34 × 10^−4^	↓ 1.77	0.141	1	=1.00
Threonine, 3TMS (RI: 1400)	0.730	9.76 × 10^−2^	=1.14	1.540	1.58 × 10^−7^	↓ 1.71
Trehalose, 8TMS (RI: 2781)	1.127	1.06 × 10^−3^	↓ 10.10	–		
Tryptophan, 3TMS (RI: 2244)	1.138	4.53 × 10^−4^	↑ 1.90	1.119	1.29 × 10^−2^	↑ 1.28
Tyrosine, 3TMS (RI: 1962)	1.293	3.24 × 10^−6^	↑ 1.81	1.323	3.28 × 10^−4^	↑ 1.21
Uracil, 2TMS (RI: 1351)	0.968	1.20 × 10^−2^	=1.67	0.901	1.05 × 10^−1^	=1.38
Valine, 2TMS (RI: 1230)	1.043	4.41 × 10^−3^	↓ 1.16	0.512	3.37 × 10^−1^	=1.05
Vitamin B6, 3TMS (RI: 1924)	0.660	7.28 × 10^−1^	=1.36	0.564	1.76 × 10^−1^	=1.09

Metabolites are listed in alphabetical order. Arrows indicate the direction (trend) of fold change comparing the two classes: (↑) upregulated in the dual-species biofilm; (↓) down-regulated in the dual-species biofilm; (=) no statistically significant change; (–) metabolite not conserved (not detected) in the two compared classes. RI represents the Kovats retention index, and TMS is the trimethylsilyl function, (CH_3_)_3_Si-.

## Data Availability

Not applicable.

## Supplementary Materials

The following are available online at https://www.mdpi.com/article/10.3390/ijms22073496/s1: Figure S1: Partial least-squares discriminant analysis (PLS-DA) score plots obtained from comparison of the *K. pneumoniae* biofilm and planktonic classes; Figure S2: Partial least-squares discriminant analysis (PLS-DA) score plots obtained from comparison of the *C. albicans* biofilm and planktonic classes; Table S1: Identified metabolites from metabolomic analysis of planktonic and biofilm cultures of *C. albicans* and *K. pneumoniae*; Table S2. Identified metabolites from metabolomic analysis of dual-species biofilm cultures of *C. albicans*/*K. pneumoniae* after 24 h of incubation compared with dual-species biofilm after 48 h of incubation; Table S3. Additional information on metabolites identified in this work.
