# Supplementary material for: GC-MS-Based Metabolomics Study of Single- and Dual-Species Biofilms of Candida albicans and Klebsiella pneumoniae
Source: Int J Mol Sci. 2021 Mar 28;22(7):3496. doi: 10.3390/ijms22073496 (PMC8037927; doi:10.3390/ijms22073496)
Supplement: Supplementary file 1 [file ijms-22-03496-s001.pdf]

Supplementary Materials

GC-MS based metabolomics study of single and dual-species biofilms of *Candida albicans* and *Klebsiella pneumoniae*

Emilia Galdiero [1], Maria Michela Salvatore [2,*], Angela Maione [1], Elisabetta de Alteriis [1], Anna Andolfi [2,3], Francesco Salvatore [2], Marco Guida [1].

[1] Department of Biology, University of Naples 'Federico II', via Cinthia, 80126, Naples, Italy; egaldier@unina.it (E.G.); angela.maione@unina.it (A.M.); dealteri@unina.it (E.d.A.); marco.guida@unina.it (M.G.)

[2] Department of Chemical Sciences, University of Naples 'Federico II', via Cinthia, 80126 Naples, Italy; andolfi@unina.it (A.A.); frsalvat@unina.it (F.S.)

[3] BAT Center—Interuniversity Center for Studies on Bioinspired Agro-Environmental Technology, University of Naples 'Federico II', Portici, 80055 Naples, Italy

*Correspondence: mariamichela.salvatore@unina.it; Tel.: +39-081674372

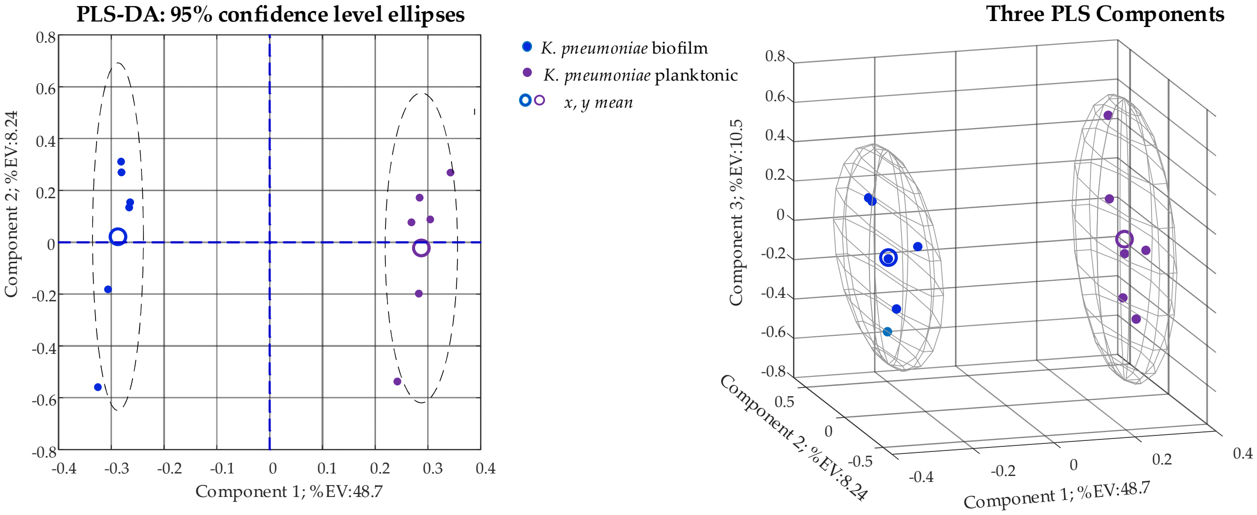

Figure S1. Partial least-squares discriminant analysis (PLS-DA) score plots obtained from comparison of *K. pneumoniae* biofilm and planktonic classes.

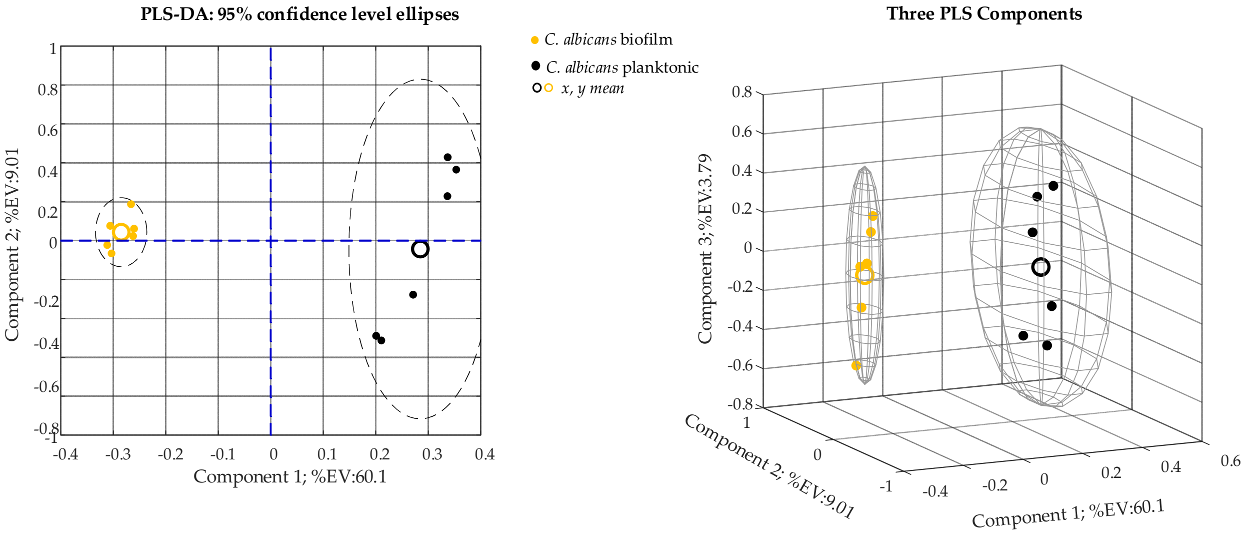

Figure S2. Partial least-squares discriminant analysis (PLS-DA) score plots obtained from comparison of *C. albicans* biofilm and planktonic classes.

Table S1. Identified metabolites from metabolomic analysis of planktonic and biofilm cultures of *C. albicans* and *K. pneumoniae*. Arrows indicate the direction (trend) of fold change comparing the two classes: (↑), up-regulated in biofilm culture; (↓), down-regulated in biofilm culture; (=) no statistically significant change; (—) indicates that the metabolite is not conserved in the two compared classes. RI represents Kovats retention index and TMS is the trimethylsilyl function, $(CH_3)_3Si$-.

	***K. pneumoniae* biofilm vs planktonic cultures**			***C. albicans* biofilm vs planktonic cultures**		
**Name**	**VIP score**	***t*-test '*p*-value'**	**Fold change**	**VIP score**	***t*-test '*p*-value'**	**Fold change**
3-Deoxy-D-arabino-hexonic acid γ-lactone, 3TMS (RI: 1797)	1.412	6.73 x 10^{-9}	↓ 2.05	1.01	2.99 x 10^{-3}	↑ 2.17
Acetamide, N,N-diethyl- (RI: 1045)		—		0.64	1.06 x 10^{-1}	= 2.64
Alanine, 2TMS (RI: 1124)	1.169	1.39 x 10^{-3}	↑ 1.39	1.16	8.17 x 10^{-5}	↑ 1.91
Arabitol, 5TMS (RI: 1750)		—		1.01	2.70 x 10^{-3}	↓ 1.75
Asparagine, 3TMS (RI: 1687)	1.335	1.11 x 10^{-5}	↑ 22.97	0.96	6.73 x 10^{-3}	=1.94
Aspartic acid, 3TMS (RI: 1540)	0.799	6.89 x 10^{-2}	= 1.42		—	
Beta-Lactic acid, 2TMS (RI: 1156)		—		0.92	9.60 x 10^{-3}	= 1.94
Butanoic acid, 3TMS (RI: 1425)	0.461	3.09 x 10^{-1}	= 1.49	0.66	9.82 x 10^{-2}	= 1.63
Citric acid, 4TMS (RI: 1844)	0.883	3.67 x 10^{-2}	= 1.88	0.51	2.72 x 10^{-1}	= 1.45
Cyclo-(Phe-pro) (RI: 2434)	1.264	1.55 x 10^{-4}	↓ 2.34	1.01	2.66 x 10^{-3}	↑ 1.76
Glutamic acid, 3TMS (RI: 1638)	1.282	8.90 x 10^{-5}	↓ 1.40	1.21	1.07 x 10^{-5}	↑ 2.17
Glyceric acid, 3TMS (RI: 1346)	1.409	1.32 x 10^{-8}	↑ 10.72	1.12	3.05 x 10^{-4}	↑ 2.58
Glycerol, 3TMS (RI: 1290)		—		0.84	2.30 x 10^{-2}	= 1.47
Glycine, 2TMS (RI: 1136)	0.832	4.87 x 10^{-2}	= 1.42	1.25	2.98 x 10^{-7}	↑ 2.48
Glycolic acid, 2TMS (RI: 1100)	1.373	1.08 x 10^{-6}	↓ 3.75	1.07	1.23 x 10^{-3}	↑ 2.17
Isoleucine, 2TMS (RI: 1307)	1.184	9.65 x 10^{-4}	↓ 1.60	1.23	4.27 x 10^{-6}	↑ 1.99
Lactic acid, 2TMS (RI: 1083)	1.006	1.11 x 10^{-2}	↑122.93	0.63	1.27 x 10^{-1}	= 1.36
Leucine, 2TMS (RI: 1286)		—		1.16	8.84 x 10^{-5}	↑ 3.62
Lysine, 3TMS (RI: 1722)	0.383	4.07 10^{-1}	= 1.07	1.13	2.33 x 10^{-4}	↑ 3.67
Methionine, 3TMS (RI: 1536)	0.577	2.33 x 10^{-1}	= 1.03	1.25	3.51 x 10^{-7}	↑ 1.50
Mevalonic acid, 3 TMS (1574)	1.430	1.39 x 10^{-12}	↓ 59.72		—	
Nicotinic acid TMS (RI: 1304)	1.395	1.22 x 10^{-7}	↓ 2.01	1.23	2.03 x 10^{-6}	↑ 1.71
Ornithine, 3TMS (RI: 1632)	1.149	1.85 x 10^{-3}	↓ 2.02		—	
Phenylalanine, 2TMS (RI: 1647)	1.365	1.88 x 10^{-6}	↓ 1.35	1.06	1.09 x 10^{-3}	↑ 1.91
Phosphate, 3TMS (RI: 1297)	0.920	2.63 x 10^{-2}	= 1.65	1.04	4.28 x 10^{-3}	↑ 716.79
Piperidine-2-carboxylic acid, 2TMS (RI: 1624)	1.366	1.69 x 10^{-6}	↓ 1.77	1.19	2.38 x 10^{-5}	↑ 2.24
Proline, 2TMS (RI: 1314)	0.420	3.72 x 10^{-1}	= 1.74	1.26	6.77 x 10^{-8}	↑ 39.10
Propanoic acid, 2methyl-2,3bishidroxy, 3TMS (RI: 1336)	0.923	2.84 x 10^{-2}	= 3.14	1.18	3.96 x 10^{-5}	↑ 5.51
Pyroglutamic acid, 2TMS (RI: 1546)	1.085	4.49 x 10^{-3}	↓ 1.24	0.43	4.00 x 10^{-1}	↑ 1.22
Pyruvic acid, 2TMS (RI: 1108)		—		0.88	2.69 x 10^{-2}	= 2.04
Shikimic acid, 4TMS (RI: 1951)	0.843	4.92 x 10^{-2}	↑ 3.08		—	
Serine, 3TMS (RI: 1375)		—		1.20	1.11 x 10^{-5}	↑ 1.50
Succinic acid, 2TMS (RI: 1322)	1.431	5.45 x 10^{-13}	↓ 16.68	1.09	7.00 x 10^{-4}	↑ 1.50
Trehalose, 8TMS (RI: 2781)		—		0.68	1.16 x 10^{-1}	= 1.50
Threonine, 3TMS (RI: 1400)	1.431	1.88 x 10^{-12}	↑ 433.33	1.22	5.06 x 10^{-6}	↑ 2.16
Tryptophan, 3TMS (RI: 2244)	1.264	1.61 x 10^{-4}	↓ 1.91	1.11	5.41 x 10^{-4}	↑ 2.40
Tyrsine, 2TMS (RI: 1962)	0.500	2.70 x 10^{-1}	= 1.35	0.97	5.85 x 10^{-3}	= 1.92
Uracil, 2TMS (RI: 1351)	1.186	9.10 x 10^{-4}	↓ 1.79	0.68	1.20 x 10^{-1}	= 1.36
Valine, 2TMS (RI: 1230)	1.262	1.66 x 10^{-4}	↓ 1.41	1.21	6.24 x 10^{-6}	↑ 2.18
Vitamin B6, 3TMS (RI: 1924)	0.694	1.12 x 10^{-1}	= 1.86	0.66	1.07 x 10^{-1}	= 1.63

Table S2. Identified metabolites from metabolomic analysis of dual biofilm cultures of *C. albicans/K. pneumoniae* after 24 h of incubation compared with dual species biofilm after 48 h of incubation. Arrows indicate the direction (trend) of fold change comparing the two classes: (↑), up-regulated in dual species biofilm after an incubation period of 24 h; (↓), down-regulated in dual species biofilm after an incubation period of 24 h; (=) no statistically significant change. RI represents Kovats retention index and TMS is the trimethylsilyl function, $(CH_3)_3Si$.

	**Dual species 24 h vs 48 h**		
**Name**	**VIP score**	***t*-test '*p*-value'**	**Fold change**
Acetamide, N,N-diethyl- (RI: 1045)	1.226	5.74×10^{-4}	↑ 3.10
Adenine, TMS (RI: 1890)	1.430	7.23×10^{-9}	↑ 13.28
Alanine, 2TMS (RI: 1124)	1.126	3.09×10^{-3}	↑ 1.27
Arabitol, 5TMS (RI: 1750)	1.082	5.46×10^{-3}	↓ 1.76
Asparagine, 3TMS (RI: 1687)	0.700	1.16×10^{-1}	= 1.12
Aspartic acid, 3TMS (RI: 1540)	1.123	3.32×10^{-3}	↓ 1.41
Beta-lactic acid, 2TMS (RI: 1156)	1.008	1.23×10^{-2}	↓ 1.41
Butanoic acid, 3TMS (RI: 1425)	0.294	5.50×10^{-1}	= 1.23
Citric acid, 4TMS (RI: 1844)	0.493	2.88×10^{-1}	= 1.11
Cyclo-(Phe-Pro) (RI: 2434)	1.376	2.81×10^{-6}	↓ 4.50
3-Deoxy-D-arabino-hexonic acid γ-lactone, 3TMS (RI: 1797)	1.432	4.64×10^{-9}	↑ 11.79
Glutamic acid, 3TMS (RI: 1638)	1.186	1.19×10^{-3}	↑ 1.22
Glyceric acid, 3TMS (RI: 1346)	0.087	9.75×10^{-1}	= 1.00
Glycerol, 3TMS (RI: 1290)	1.000	1.34×10^{-2}	= 1.42
Glycine, 2TMS (RI: 1136)	0.455	3.26×10^{-1}	= 1.13
Isoleucine, 2TMS (RI: 1307)	0.494	2.79×10^{-1}	= 1.81
2-Isopropyl-3-ketobutyrate, 2TMS (RI: 1463)	1.451	1.2×10^{-16}	↓ 15.21
Leucine, 2TMS (RI: 1286)	0.498	2.88×10^{-1}	= 1.69
Lysine, 3TMS (RI: 1722)	1.406	2.52×10^{-7}	↑ 6.42
Methionine, 2TMS (RI: 1536)	1.333	2.51×10^{-5}	↑ 1.58
2-Methylglutaconic acid (3TMS) (RI: 1590)	1.431	5.66×10^{-9}	↓ 5.81
Nicotinic acid, TMS (RI: 1304)	0.979	1.62×10^{-2}	= 1.17
Ornithine, 3TMS (RI: 1632)	1.399	4.85×10^{-7}	↑ 32.09
Phenylalanine, 2TMS (RI: 1647)	1.031	9.74×10^{-3}	↑ 1.15
Phosphate, 3TMS (RI: 1297)	0.618	1.74×10^{-1}	= 1.20
Piperidine-2-carboxylic acid (2TMS) (RI: 1624)	0.569	2.19×10^{-1}	= 1.08
Proline, 2TMS (RI: 1314)	1.122	3.26×10^{-3}	↓ 3.48
Propanoic acid, 2methyl-2,3bishidroxy, 3TMS (RI: 1336)	0.532	2.48×10^{-1}	= 1.58
Pyroglutamic acid, 2TMS (RI: 1546)	0.233	6.43×10^{-1}	= 1.10
Pyruvic acid, 2TMS (RI: 1108)	0.281	5.49×10^{-1}	= 1.14
Succinic acid, 2TMS (RI: 1322)	1.387	1.41×10^{-6}	↓1.35
Threonine, 3TMS (RI: 1400)	1.402	3.97×10^{-7}	↓ 1.49
Trehalose, 8TMS (RI: 2781)	1.199	9.70×10^{-4}	↓ 3.08
Tryptophan, 3TMS (RI: 2244)	1.388	5.46×10^{-6}	↑ 4.37
Tyrosine, 3TMS (RI: 1962)	1.384	1.66×10^{-6}	↑ 2.12
Uracil, 2TMS (RI: 1351)	0.890	3.40×10^{-2}	↓ 1.37
Valine, 2TMS (RI: 1230)	0.753	8.75×10^{-2}	↑ 1.05
Vitamin B6, 3TMS (RI: 1924)	1.246	3.52×10^{-4}	↓ 4.35

Table S3. Additional information on metabolites identified in this work. MF represents the so-called Match Factor calculated by the dot-product function that measures the cosine of the angle between spectra represented as vectors. MF = 1000 correspond to a perfect match between a pair of compared spectra. RI_{exp} and RI_{DB} represent, respectively, Kovats programmed temperature retention indices evaluated in this work (on a 5% phenyl methyl poly siloxane) and gathered from literature and databases (for identical or similar) stationary phases. %ΔRI is the percent difference between experimental and literature RI values. TMS is the trimethylsilyl function, $(CH_3)_3Si$.

**Name**	**MF**	**RI_{exp} (this work)**	**RI_{DB} (literature)**	**%ΔRI**
Acetamide, N,N-diethyl-	877	1045	–	–
Adenine, TMS	896	1890	1898	-0.42
Alanine, 2TMS	891	1124	1087	3.29
Arabitol, 5TMS	871	1750	1760	-0.57
Asparagine, 3TMS	899	1687	1666	1.24
Aspartic acid, 3TMS	852	1540	1513	1.75
Beta-lactic acid, 2TMS	754	1156	1151	0.43
Butanoic acid, 3TMS	805	1425	1431	-0.42
Citric acid, 4TMS	839	1844	1839	0.27
Cyclo-(Phe-Pro)	808	2434	–	–
3-Deoxy-D-arabino-hexonic acid γ-lactone, 3TMS	934	1797	1771	1.45
Glutamic acid, 3TMS	890	1638	1647	-0.55
Glyceric acid, 3TMS	899	1346	1342	0.30
Glycerol, 3TMS	833	1290	1292	-0.16
Glycine, 2TMS	933	1136	1111	2.20
Glycolic acid (2TMS)	841	1100	1078	2.00
Isoleucine, 2TMS	934	1307	1300	0.54
2-Isopropyl-3-ketobutyrate, 2TMS	755	1463	–	–
Lactic acid, 2TMS	960	1083	1065	1.66
Leucine, 2TMS	763	1286	1287	-0.08
Lysine, 3TMS	947	1722	1703	1.10
Methionine, 2TMS	905	1536	1531	0.33
2-Methylglutaconic acid, 3TMS	774	1590	–	–
Mevalonic acid, 3 TMS	886	1574	1572	0.13
Nicotinic acid, TMS	841	1304	1301	0.23
Ornithine, 3TMS	901	1632	1612	1.23
Phenylalanine, 2TMS	869	1647	1629	1.09
Phosphate, 3TMS	717	1297	1282	1.16
Piperidine-2-carboxylic acid, 2TMS	826	1624	1505	7.33
Proline, 2TMS	885	1314	1316	-0.15
Propanoic acid, 2methyl-2,3bishidroxy, 3TMS	828	1336	1348	-0.90
Pyroglutamic acid, 2TMS	910	1546	1521	1.62
Pyruvic acid, 2TMS	883	1108	1090	1.62
Serine, 3TMS	893	1375	1388	-0.95
Shikimic acid, 4TMS	899	1951	1812	7.12
Succinic acid, 2TMS	942	1322	1318	0.30
Threonine, 3TMS	957	1400	1377	1.64

Trehalose, 8TMS	911	2781	2756	0.90
Tryptophan, 3TMS	945	2244	2218	1.16
Tyrosine, 3TMS	933	1962	1960	0.10
Uracil, 2TMS	758	1351	1346	0.37
Valine, 2TMS	877	1230	1234	-0.33
Vitamin B6, 3TMS	937	1924	1908	0.83
